# Tracking Objects Based on Multiple Particle Filters for Multipart Combined Moving Directions Information

**DOI:** 10.1155/2020/8839725

**Published:** 2020-12-16

**Authors:** Ngo Duong Ha, Ikuko Shimizu, Pham The Bao

**Affiliations:** ^1^Faculty of Mathematics and Computer Science, University of Science, Vietnam National University-Ho Chi Minh City, Ho Chi Minh, Vietnam; ^2^Information Technology Faculty, Ho Chi Minh City University of Food Industry, Ho Chi Minh, Vietnam; ^3^Tokyo University of Agriculture and Technology, Tokyo, Japan; ^4^Information Science Faculty, Sai Gon University, Ho Chi Minh, Vietnam

## Abstract

Object tracking is an important procedure in the computer vision field as it estimates the position, size, and state of an object along the video's timeline. Although many algorithms were proposed with high accuracy, object tracking in diverse contexts is still a challenging problem. The paper presents some methods to track the movement of two types of objects: arbitrary objects and humans. Both problems estimate the state density function of an object using particle filters. For the videos of a static or relatively static camera, we adjusted the state transition model by integrating the movement direction of the object. Also, we propose that partitioning the object needs tracking. To track the human, we partitioned the human into *N* parts and, then, tracked each part. During tracking, if a part deviated from the object, it was corrected by centering rotation, and the part was, then, combined with other parts.

## 1. Introduction

The object tracking in videos is a technique that has many applications in many fields. For example, in the biomedical field [1, 2], the object-tracking technique is applied to automatically track cells while they are born, duplicated, or as they move and die. Another example is the application of the technique in autopilot systems, where it is used to observe and track the vehicles around the driving car [3, 4] or footballer tracking [5–7]. A highly accurate vehicle-tracking program is an indispensable necessity for safety. Moreover, the tracking technology is usually combined with the identification and recognition systems to create a complete tactic for real-life applications.

Tracking objects in video is difficult due to many challenges that all are needed to be considered and solved. The first challenge is that we do not know, in advance, the object that we need to track. There may be no information about that object. In the absence of information, the object description for the program script must be highly general. Another challenge is that the tracked object has heterogeneity in colors, which vary by each part of the object. For example, to track human movement, the head is characterized by the hair color (black and yellow), while the body and legs are described by the color of the shirt and pants that the person is wearing. Because of the challenges and difficulties mentioned above, no comprehensive tracking algorithm can be adopted for all problems.

In this paper, we present the method to modify the state model according to the direction of predicting that an object appears in the same direction of motion with a higher probability. In addition, we explore the effectivity to track partially obscured objects by tracking its visible sections. To do this, we divided the object into multiple sections and tracked these sections independently. When some parts obscure the object, our approach should still successfully track the object movement. We also present the experimental particle filter model and present a suggestion for integrating information on the direction of the object's movement, the *N*-particle filter model, to track each part then combines.

The rest of the paper is organized as follows: The most relevant work that motivated this paper is reviewed in Section 2.  Section 3 describes, in detail, our method multiple particle filters for multipart combined moving directions information.  Section 4 summarizes the results from our method.  Section 5 is the discussion of our paper.

## 2. Related Work

The correlation filters approach is a powerful tool in digital signal processing [8, 9]. This algorithm class utilizes the properties of Fourier transform of turning convolution in the spatial domain into function multiplication in the Fourier domain [10–13]. The original idea of the correlation filter was to solve the problem of locating an object in an image. In other words, if the object of interest appears in the image, its position including the axis coordinates is determined. The tool to solve this problem is the average synthetic exact filter [10]. The next, correlation filter, is the Total Minimum Output Error, studied by Bolme et al. [13]. This tracking method is very powerful and can cope with situations such as changing light and changing the size and shape of objects.

Aviden et al. [14] at Misubishi Electric Research Labs considered object tracking as the binary classification problem to distinguish background pixels and tracking-object pixels using the AdaBoost technique. The method's idea was training weak classification functions to classify the background and object and, then, combine them to form a strong classification based on the Adaboost mechanism. But, the author realized that if an object is not in the rectangle form, pixels in the containing object rectangle but not in the object will be labeled as belonging to the object. These pixels are considered alien elements, while AdaBoost is sensitive to alien elements [15]. In addition, some other limitations of the approach are as follows: it has not solved the completely obstructed object's situation in a long time and the featured space used in the algorithm does not yet utilize the spatial information of the image.

The approach based on random process filtering has been studied for a long time in the field of mathematical statistics, and there have been many discovered impressive results [16–18]. Most of the algorithms based on this approach are based on the Bayes optimal solution for the hidden Markov filtering problem [19–21]. That means building a hidden Markov model plays a key role, and the model is more accurate; in fact, more Bayesian solutions accurately estimate the state of the object. The work in [20] uses the featured color histogram to construct a particle filter to track objects. The work in [22] uses gentle AdaBoost to construct an updated observation model over time.

Recently, the Siamese network-based trackers have received significant attention for their well-balanced tracking accuracy and efficiency. These trackers formulate visual tracking as a cross-correlation problem and are expected to better leverage the merits of deep networks from end-to-end learning [23–30]. Bhat et al. [31] proposed a gradient-guided method to update the template. Li et al. [32] developed a discriminative model-prediction architecture for tracking.

## 3. Methodology

**Problem 1 .**

Highlight that first frame coordinates (*x*_1_, *y*_1_, *ω*_1_, *h*_1_) are given and we need to infer object coordinates (*x*_*k*_, *y*_*k*_, *ω*_*k*_, *h*_*k*_) in the subsequent frames.

Filtering the state (*x*_*k*_, *y*_*k*_, *ω*_*k*_, *h*_*k*_) of the object in the next frames, we rely on the hidden Markov model theory with the construction of two models: state transitions and observations. The state transitions model in the studies is quite similar and are all Gaussian motion, and the main difference in the algorithms depends on the observed model. Using particle filters allows us to better handle color clutter in the background, as well as tracking completely obstructed objects.

**Figure 1:**
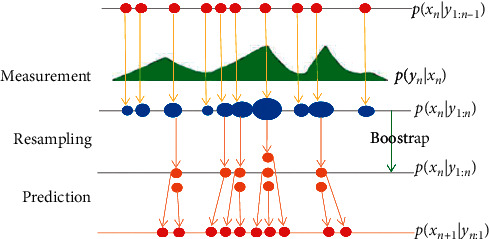
Demonstrate the operating mechanism of a particle filter.

The principle of the particle filter according to the Figure 1, including 3 steps:

  Measurement: calculating the samples' weights based on the observation at time *n*
  Resampling: resampling or fine tuning (based on the threshold of the return weights) the samples to remove or adjust the samples in which the object positions are overmismatched at the current time
  Prediction: predicting the object status at time *n* + 1 based on the likelihoods from time *n* to the previous states

Based on the particle filter operating mechanism in Figure 1, we present the approximate results of posterior density function *p*(*x*_*k*_*|y*_1:*k*_) at time *k*.

From the posterior distribution at time *k* – *1*, *p*(*x*_*k*_*|y*_1:*k*−1_), we calculate the prior distribution for the time *k* (without observing *y*_*k*_) by using Chapman–Kolmogorov equality [16], *p*(*x*_*k*_*|y*_1:*k*−1_)=∫*p*(*x*_*k*_*|x*_*k*−1_).*p*(*x*_*k*−1_*|y*_1:*k*−1_)d*x*_*k*−1_, where *p*(*x*_*k*_*|x*_*k*−1_) already exists in the state transition model and *p*(*x*_*k*−1_*|y*_1:*k*−1_) is the posterior root of step *k* − *1*.

After observing *y*_*k*_, we update the prior density function at the predicted step at the level *k*: *p*(*x*_*k*_*|y*_1:*k*_)=(*p*(*y*_*k*_*|x*_*k*_)^*∗*^*p*(*x*_*k*_*|y*_1:*k*−1_)/*p*(*y*_*k*_*|y*_1:*k*−1_)), where *p*(*y*_*k*_*|x*_*k*_) already exists in the observation model and *p*(*x*_*k*_*|y*_1:*k*−1_) is an a priori at the time *k* calculated in the previous step.

As a result, we obtain a weighted pattern representing the posterior density function at the time *k*: *p*(*x*_*k*_*|y*_1:*k*_) ~ {*x*_*k*_^*l*^, (1/*Z*)*ω*_*k*−1_^*l∗*^*p*(*y*_*k*_*|x*_*k*_^*l*^)}_*l*=1_^*L*^.

When an object is in motion, it usually moves in a specific trajectory. Therefore, to predict the object's location, we propose integrating the direction of motion, which will be discussed in detail in Section 3.1. In addition, different parts of an object carry their distinctive characteristics of shape, color, light absorption, and reflection capacity. If we use a particle filter, it can yield false tracking results. Besides, if a part of the object has the same color and brightness level as any other object in the frame, the tracking may be distorted. To fix this problem, we propose to divide the object into many parts, each of which will have the same properties. We, then, track the movement of each part with the constraints that these parts move in the same direction and maintain similar area and shape.

### 3.1. Moving Direction Information

With the videos filtered out from the dataset in which the camera was relatively stable, we modified the state transition model in the hidden Markov model by integrating the direction of the object movement. This means that instead of using the Gaussian motion state model, we projected these Gauss functions into several different directions with different ratios before we made a new pattern. Because each object moves in a specific trajectory, the direction of the object's motion will remain constant for a certain period of time. Specifically, we consider the direction of motion as a separate component. At each assessment, we will update the direction of motion. We use this direction of motion to impact the particle filter at the prediction step, with the purpose that the particle filter will predict the object appearing in the same direction with higher probability.

$\overset{⟶}{v}$ is rotated an angle *θ* by multiplying the rotation matrix by $\overset{⟶}{v}$ as follows:

(1) $$\begin{array}{c} \overset{⟶}{v_{\theta }}=\left(\begin{array}{cc} \cos\;\theta & -\sin\;\theta \\ \sin\;\theta & \cos\;\theta \end{array}\right).\overset{⟶}{v}, \end{array}$$

where *θ*={0, *π*/4, −*π*/4, *π*/2, −*π*/2, 3*π*/4, −3*π*/4, *π*}.

**Figure 2:**
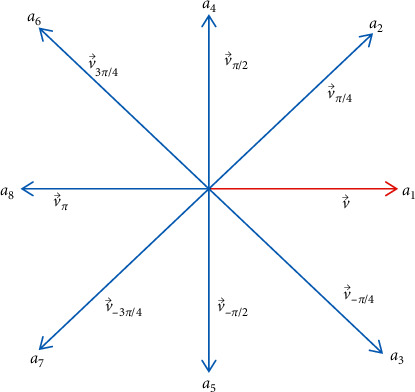
Distribute probabilities for moving directions.

Figure 2 depicts the probability distribution in the direction of the object's appearance, in which the red direction (in the direction of *a*_1_) is the predicted movement direction of the object, *a*_1_, *a*_2_, *a*_3_, *a*_4_, *a*_5_, *a*_6_, *a*_7_, *a*_8_ are real numbers in [0, 1], and ∑_*i*=1_^8^*a*_*i*_=1. In the prediction step, we translate *N*_*s*_ · *a*_1_ particle by $\overset{⟶}{v}$, *N*_*s*_ · *a*_2_ particle by $\overset{⟶}{v_{\left(\pi /4\right)}}$,*N*_*s*_ · *a*_3_ particle by $\overset{⟶}{v_{\left(-\pi /4\right)}}$, *N*_*s*_ · *a*_4_ particle by $\overset{⟶}{v_{\left(\pi /2\right)}}$, *N*_*s*_ · *a*_5_ particle by $\overset{⟶}{v_{\left(-\pi /2\right)}}$, *N*_*s*_ · *a*_6_ particle by $\overset{⟶}{v_{\left(3\pi /4\right)}}$, *N*_*s*_ · *a*_7_ particle by $\overset{⟶}{v_{\left(-3\pi /4\right)}}$, and *N*_*s*_ · *a*_8_ particle by $\overset{⟶}{v_{\pi }}$.

We propose the particle filter algorithm to integrate the direction of motion in Algorithm 1.

### 3.2. Multiple Particle Filters Model

#### 3.2.1. Multiparts of an Object

**Figure 3:**
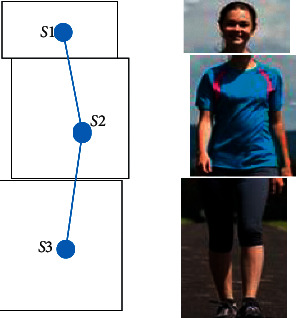
Some structure II partition objects.

While considering the problem where the object shapes are less changing, if the object includes many parts with dissimilarity about the colors and contrast, using 1 particle filter for tracking will lead to incorrect tracking. Therefore, an object needs to separate each area with similar color, grayscale, and contrast into *n* parts, each part being tracked separately. In this way, the parts which affected by the environment and other object artifacts will cause incorrect position identification which will need to be adjusted. For example, a human object can be normally represented by a 3-partition structure as illustrated in Figure 3. This structure divides the human object based on the gray color changes among the black head, the white shirt body, and the black pants legs. The resulting human object will be divided into 3 parts with a border represented by a different gray level, each part using a particle filter to track and combine based on the best feature matching part.

#### 3.2.2. Build Model

The adjustment of deviated areas should take two steps:

- The center of the similar areas changed, which allowed the incorrect position of the similar areas to be adjusted accordingly to the correct position of the object
- Size ratio of similar area allowed similar areas to scale the height to the height of the original object

Thus, when one part of the object is obscured or similar to another, we can restore and track enough.

#### 3.2.3. Fine Tuning Parts

Once the anterior root of the object is defined, the object is defined into *n* parts in a structure H, as shown in Figure 3. We, then, used one particle filter to track each part *S*_1_, *S*_2_,…, *S*_*n*_. At each time data point, each particle filter in the tracking area can diverge from each other. Therefore, a modification model is needed to correct this issue. We used the collected assessment data of each part to evaluate which tracked part behaves the best. We kept this best-tracked part fixed and applied the rotation algorithm to *n* − 1 other parts using the fixed part as the origin. The adjustment of *N*-particle filters when tracking an object in the frame time *k* is described below.

  Step 1: we calculate the rotation from the center of each section with the remaining *n* − 1 based on frame 0.

(2) $$\begin{array}{c} \theta_{ij}^{0}=\left(S_{i}^{0},S_{j}^{0}\right),\text{where }i,j=\bar{1,n}. \end{array}$$

**Figure 4:**
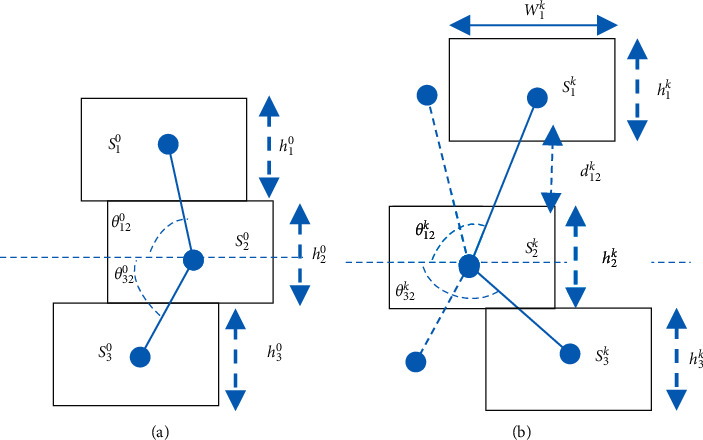
(a) The angle *θ*_12_^0^, *θ*_32_^0^ is the angle between the center *S*_2_^0^ and the center *S*_1_^0^, *S*_3_^0^ based on Frame 0, and *h*_1_^0^, *h*_2_^0^, *h*_3_^0^ are the heights of *S*_1_^0^, *S*_2_^0^, *S*_3_^0^. (b) In the *k*th frame, the parts *S*_1_^*k*^, *S*_3_^*k*^ have been skewed from *S*_2_^*k*^ with angle *θ*_12_^*k*^, *θ*_32_^*k*^, and *h*_1_^*k*^, *h*_2_^*k*^, *h*_3_^*k*^ are the heights of *S*_1_^*k*^, *S*_2_^*k*^, *S*_3_^*k*^.

  For example, the angle of rotation from the center of *S*_2_^0^ compared to the rest of *S*_1_^0^, *S*_3_^0^ is the angle *θ*_12_^0^, *θ*_32_^0^ according to Figure 4(a).
 Step 2 : we suppose that *S*_1_^*k*^, *S*_2_^*k*^,…, *S*_*n*_^*k*^ are estimates and *S*_1_^0^, *S*_2_^0^,…, *S*_*n*_^0^ are parts of the object in the original image. The distance is calculated as ${\text{distance}}_{i}={\left\|\text{HOG}\left(S_{i}^{k}\right)-\text{HOG}\left(S_{i}^{0}\right)\right\|}^{2},\;i=\bar{1,n}$.
  However, because the division of parts may not be equal, to determine which is best, we multiply the coefficient by each distance and, then, compare *K*_1_*∗*distance_1_, *K*_2_*∗*distance_2_,…, *K*_*n*_*∗*distance_*n*_. The smallest value is considered the best estimate. The best estimate is placed at the *k*th frame as *S*_min_^*k*^.
 Step 3 : when the best part (*S*_min_^*k*^) was selected, we performed the center rotation of the remaining *n* − 1 relative to the best part (*S*_min_^*k*^) with the rotation angle defined. The result is the new center coordinates of the *n* − 1 part.
  find the new center coordinates of the part *S*_*i*_^*k*^ with $i=\bar{1,\left(n-1\right)}$ by calculating the center rotation of the section *S*_*i*_^*k*^ compared to the center of the part *S*_min_^*k*^ with the angle of rotation (*θ*_*i*min_^*k*^ − *θ*_*i*min_^0^) as follows:

(3) $$\begin{array}{c} \overset{⟶}{v_{S_{i^{\prime }}^{k}}}=\left(\begin{array}{cc} \cos\left(\theta_{i\min}^{k}-\theta_{i\min}^{0}\right) & -\sin\left(\theta_{i\min}^{k}-\theta_{i\min}^{0}\right)\\ \sin\left(\theta_{i\min}^{k}-\theta_{i\min}^{0}\right) & \cos\left(\theta_{i\min}^{k}-\theta_{i\min}^{0}\right) \end{array}\right).{\overset{⟶}{v}}_{S_{i}^{k}}, \end{array}$$

  where ${\overset{⟶}{v}}_{S_{i}^{k}}$ is the center coordinate of the part *S*_*i*_^*k*^ and $\overset{⟶}{v_{S_{i^{\prime }}^{k}}}$ is the new center coordinate of the part *S*_*i*_^*k*^ after performing the rotation.
  For example, according to Figure 4(b), the new center coordinates of *S*_1_^*k*^, *S*_3_^*k*^ parts are found compared to *S*_2_^*k*^ centers with the rotation angle (*θ*_12_^*k*^ − *θ*_12_^0^) and (*θ*_32_^*k*^ − *θ*_32_^0^) as

(4) $$\begin{array}{c} \overset{⟶}{v_{S_{1^{\prime }}^{k}}}=\left(\begin{array}{cc} \cos\left(\theta_{12}^{k}-\theta_{12}^{0}\right) & -\sin\left(\theta_{12}^{k}-\theta_{12}^{0}\right)\\ \sin\left(\theta_{12}^{k}-\theta_{12}^{0}\right) & \cos\left(\theta_{12}^{k}-\theta_{12}^{0}\right) \end{array}\right).{\overset{⟶}{v}}_{S_{1}^{k}},\\ \overset{⟶}{v_{S_{3^{\prime }}^{k}}}=\left(\begin{array}{cc} \cos\left(\theta_{32}^{k}-\theta_{32}^{0}\right) & -\sin\left(\theta_{32}^{k}-\theta_{32}^{0}\right)\\ \sin\left(\theta_{32}^{k}-\theta_{32}^{0}\right) & \cos\left(\theta_{32}^{k}-\theta_{32}^{0}\right) \end{array}\right).{\overset{⟶}{v}}_{S_{3}^{k}}. \end{array}$$

 Step 4 : translating the particles progress of part *S*_*i*_^*k*^ with $i=\bar{1,\left(n-1\right)}$ which differs from the best part (*S*_min_^*k*^) to the position of n-1 new part (*S*_*i*′_^*k*^) is created out through Step 3.
  Step 5: the sizes of *S*_1_^*k*^, *S*_2_^*k*^,…, *S*_*n*_^*k*^ parts are scaled according to the ratio of *S*_1_^0^, *S*_2_^0^,…, *S*_*n*_^0^. That is, we will calculate *h*_*i*_^*k*^ and *w*_*i*_^*k*^ with $i=\bar{1,\left(n-1\right)}$ of the sections *S*_1_^*k*^, *S*_2_^*k*^,…, *S*_*n*_^*k*^.
  First, we find the horizontal dimensions of each *S*_*i*_^*k*^ part as follows: *w*_*i*_^*k*^=∑_*i*=1_^*n*^*w*_*i*_^*k*^/*n* with $i=\bar{1,\left(n-1\right)}$.
  Next, we find the height dimensions of each *S*_*i*_^*k*^ part as follows: *h*_*i*_^*k*^=(*w*_*i*_^*k*^*∗h*_*i*_^0^/*w*_*i*_^0^).
 Step 6 : translating the particles progress of part *S*_*i*_^*k*^ with $i=\bar{1,\left(n-1\right)}$ compared to the best part (*S*_2_^*k*^) with distance *d*_*i*min_^*k*^.

For example, as shown in Figure 4(b), the particles of *S*_1_^*k*^ are translated towards the best part (*S*_2_^*k*^) with about *d*_12_^*k*^.

We propose the multiparticle filter algorithm in Algorithms 2 and 3.

## 4. Experiment

### 4.1. Environment

Installation environment: we experiment on computers using the Windows 10 Pro 64 bit, RAM 8 GB, Chip Intel Core (TM) 5i-3210M CPU @ 2.5GHz; Matlab programming language version R2016a.

### 4.2. Data Set

In 2013, Wu et al. [33] gathered many video sources related to the track and proceeded to create ground truth for these videos to form the TB-100 dataset. Because the TB-100 is a compilation of data from many sources, the context of the videos is also very different and diverse in attributes such as the type of objects to track, color or black-and-white videos, and still or dynamic cameras. The video datasets used to support the findings of this study have been deposited in http://www.visual-tracking.net.

Challenges in the dataset include the following:

  IV- illumination variation: the brightness of the subject varies significantly
  SV- scale variation: the ratio of the rectangle containing the first image object to the current image is out of range [(1/*t*_*s*_), *t*_*s*_], *t*_*s*_ > 1(*t*_*s*_=2)
  OCC- occlusion: the object is partially or completely obscured
  DEF- deformation: nonsolid objects that change shape
  MB- motion blur: the subject is blurry due to camera movement
  FM- fast motion: groundtruth motion is greater than *t*_*m*_ pixels (*t*_*m*_ = 20)
  IPR- in-plane rotation: objects rotate in the image domain
  OPR- out-of-plane rotation: object out of the image domain
  OV- out of view: part of the object out of the image domain
  BC- background clutters: the background near the object has the same color or line as the object
  LR- low resolution: the number of pixels in the rectangle that contains the object (considering ground truth) is less than *t*_*r*_ (*t*_*r*_ = 400)

**Figure 5:**
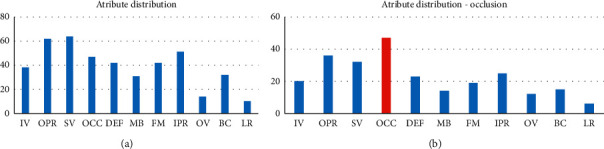
(a) Distribution of the properties throughout the data set. (b) Distribution of attributes in videos that have OCC attributes.

The abovementioned challenges are distributed in the data set, which is shown in Figure 5.

### 4.3. Evaluating

We use the evaluation criteria presented at the site [33] to evaluate the tracking algorithm.

**Figure 6:**
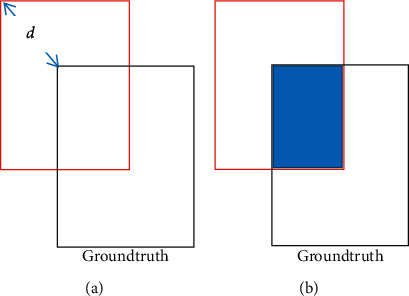
(a) Euclid distance measurement. (b) Measure of the level of overlap.

  Method 1 (*R*_1_): evaluation based on the Euclid distance (precision plot): we measure the distance Euclid *d* from the estimated center of the algorithm to the actual center of the object (ground truth), if *d* is less than or equal to a threshold *t*_0_. The view is successful according to Figure 6(a).
  Method 2 (*R*_2_): evaluation based on levels of overlap (success plot): the number of overlapping points is defined as =(|*r*_*t*_∩*r*_*a*_|/|*r*_*t*_ ∪ *r*_*a*_|), in which *r*_*t*_ is the bounding rectangle determined by the algorithm and r_a_ is the ground truth rectangle according to Figure 6(b).

We calculate the ratio of *R*_1_, *R*_2_ by (the number of successful images/the total number of images of *images*).

### 4.4. Result

The results of tracking people with the camera do not fluctuate much, the rotation angle is conserved, and the proportions on the body of people and people are not too small.

**Figure 7:**
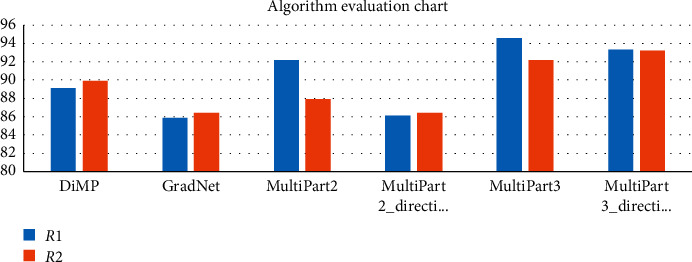
Algorithm evaluation chart.

We named Program 1 as MultiPart3 using the MultiPart algorithm by dividing the object into 3 parts in a ratio of 1 : 5 : 3; Program 2 is MultiPart3_direction using the MultiPart algorithm to calculate the direction of moving objects by dividing the object into 3 parts in a ratio of 1 : 5 : 3; Program 3 is MultiPart2 using the MultiPart algorithm by dividing the object into 2 parts in a ratio of 5 : 3; Program 4 is MultiPart2_direction using the MultiPart algorithm to calculate the direction of object movement by dividing the object into 2 parts in a ratio of 5 : 3. The abovementioned five programs compared with DiMP algorithms [31] and GradNet [32] are shown in Table 1 and Figure 7.

The MultiPart2 algorithm uses 2 particle filters in a ratio of 5 : 3 to track, allowing a large portion of the head (head and body) to be more informative, less changing over time, and “denser” than the leg. The average accuracy result (*R*1 = 92.2%, *R*2 = 87.9%) is slightly larger than that of the GradNet algorithm (*R*1 = 85.9%, *R*2 = 86.3%). With the abovementioned results, we can see that the tracking part has much information for good average results compared with the object tracking. However, for data (Dancer và Dancer2) that have a tracking object who wears a skirt or long skirt covering feet, tracking using 2 particle filters at a ratio of 5 : 3 gives a low result compared to an object trace. By tracking, the object intact in this case is best.

For the videos mentioned above, the MultiPart3 algorithm divides 3 parts in a 1 : 5 : 3 ratio, after each image has adjustments of the parts according to the rotation technique based on the structure H to bring the wrong parts to the right area of ground truth. The results of the MultiPart3 algorithm (*R*1 = 94.6% and *R*2 = 92.2%) are better than those of DiMP (*R*1 = 89.1%, *R*2 = 89.9%) and GradNet (*R*1 = 85.9%, *R*2 = 86.3%). In addition, the algorithm MultiPart3_direction is the combination of the object movement direction for average accuracy results (*R*1 = 93.3%, *R*2 = 93.2%), and this result is better than DiMP and GradNet algorithm's result. The MultiPart3_direction algorithm approximates the average accuracy result with the MultiPart3 algorithm because the dancer data have the human object that jumps up and down suddenly and the refined data take a number of frames so that the determination of the motion direction is wrong.

## 5. Conclusions

This paper presented several methods for object tracking in the videos mainly related to particle filters. To solve the general problem, we built a hidden Markov model and applied particle filters. For tracking human videos in normal condition where the human scale is preserved, we used 3 particle filters to track each part of the body or track the part of the body containing the most information, which will, then, infer to the whole body. Experimental results show dividing the object into (*n* + *m*) parts, even when *n* parts of objects are partially obscured and the remaining *m* parts are tracked normally and do not affect the tracking of the subject in the video.

The development direction of this paper is to change the observation model. We found that the gentle Adaboost training process is time consuming. However, the algorithms using correlation filters have the advantage of being fast and highly accurate. For future studies, we suggest integrating the correlation filters into the observation model to shorten the execution time. In addition, we are planning to study parts of the traced objects in parallel to shorten the execution time.

## Figures and Tables

**Figure 8:**
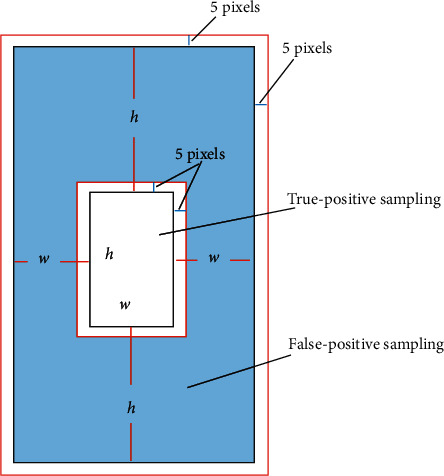
Limitations for false-positive and true-positive sampling [19].

**Algorithm 1:**
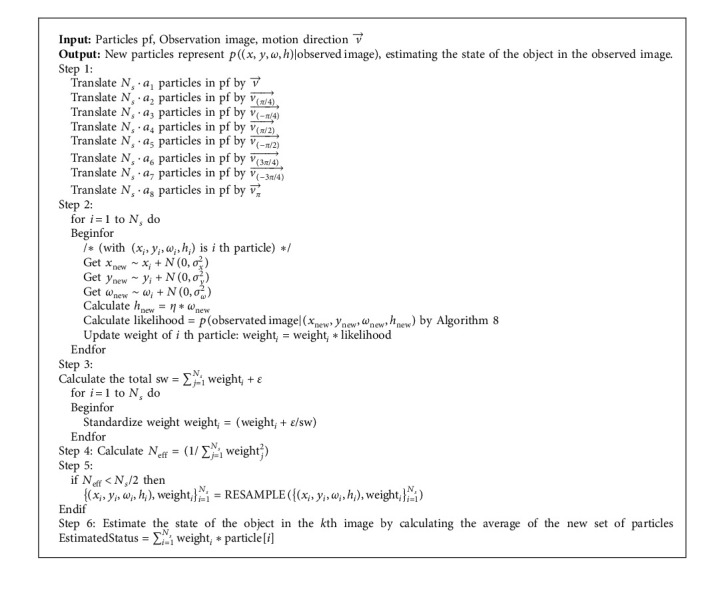
Particle filter integrated motion direction.

**Algorithm 2:**
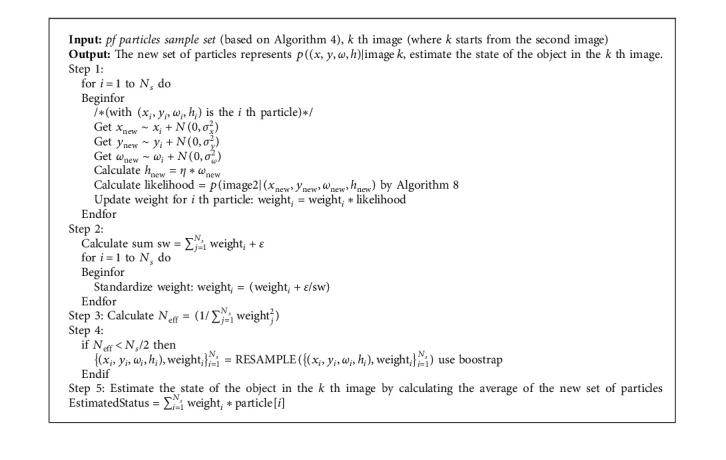
Particle filter for random processes (*x*_*n*_, *y*_*n*_, *ω*_*n*_, *h*_*n*_).

**Algorithm 3:**
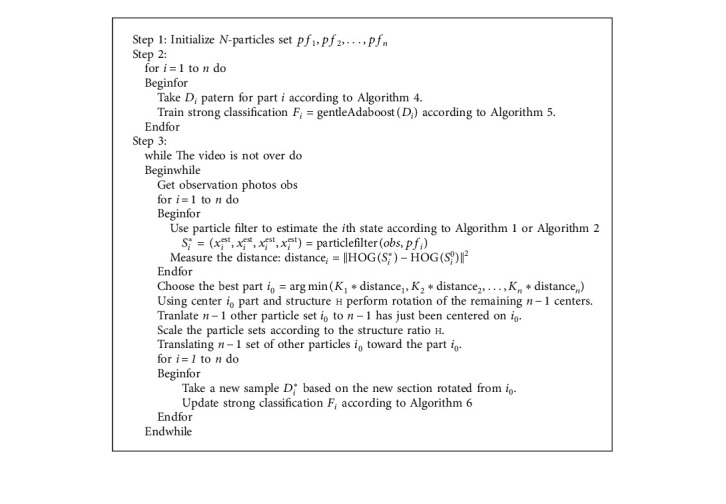
(MultiPart).

**Algorithm 4:**
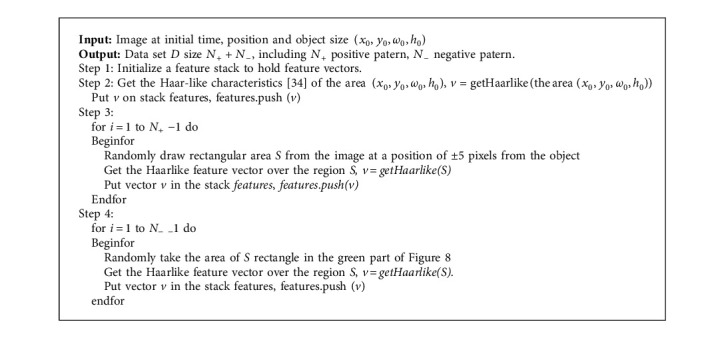
Sample to train gentle Adaboost.

**Algorithm 5:**
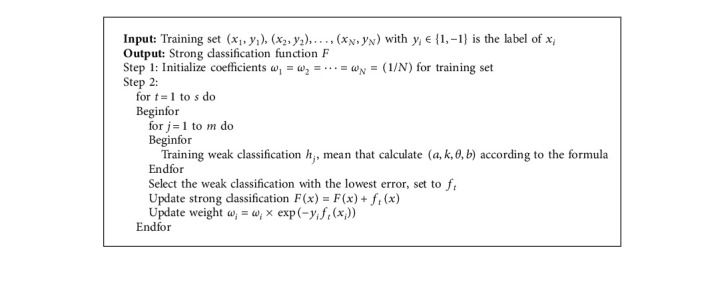
Gentle Adaboost [35].

**Algorithm 6:**
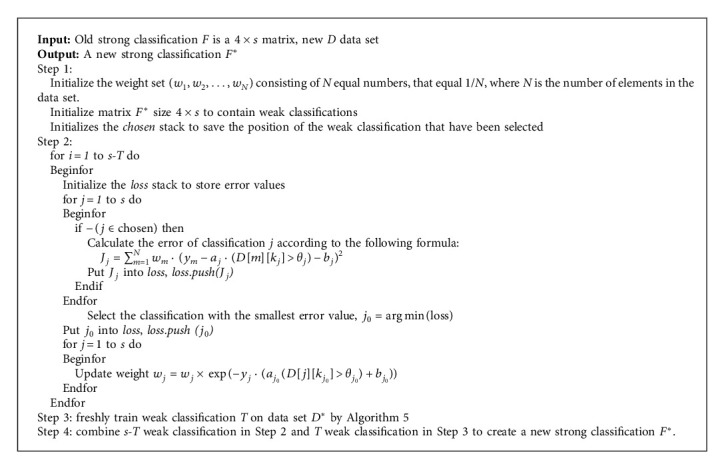
Update gentle Adaboost.

**Algorithm 7:**
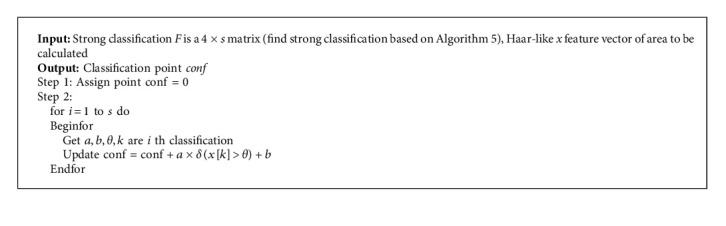
Calculate the Classification point.

**Algorithm 8:**
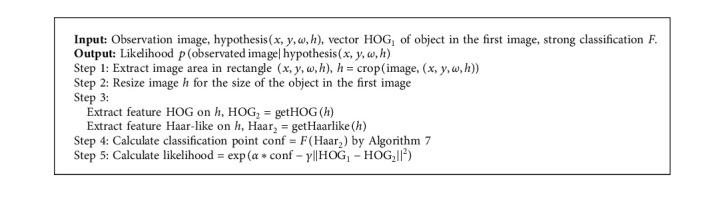
Calculate the likelihood of the image on the hypothesis of the state of the object.

**Table 1:** Algorithm results of DiMP, GradNet, MultiPart3, MultiPart3_direction, MultiPart2, and MultiPart2_direction.

Video	DiMP (%)		GradNet (%)		MultiPart2 (%)		MultiPart2_direction (%)		MultiPart3 (%)		MultiPart3 direction (%)	
	*R*1	*R*2	*R*1	*R*2	*R*1	*R*2	*R*1	*R*2	*R*1	*R*2	*R*1	*R*2
Crossing	100	100	100	100	100	98.3	100	93.3	100	100	100	100
Dancer	93.3	100	92.4	98.2	95.1	88.9	91.1	88.4	83.1	86.2	64.9	74.2
Dancer2	97.3	99.3	100	100	77.3	59.0	47.3	57.3	100	98.7	96.0	96.7
David3	90.9	90.9	100	100	73.0	81.4	64.3	67.9	80.6	84.5	92.5	92.5
Human8	100	100	8.6	7.0	100	89.1	100	99.2	100	81.3	100	89.8
Walking	100	99.8	100	99.3	100	98.5	100	98.8	99.8	99.0	100	99.3
Walking2	42.2	39.6	100	100	100	100	100	100	98.8	96.0	100	100
	89.1	89.9	85.9	86.4	92.2	87.9	86.1	86.4	94.6	92.2	93.3	93.2

**Table 2:** Notation table.

Symbol	Explanation
(*x*_*k*_, *y*_*k*_, *ω*_*k*_, *h*_*k*_)	The state of the object in the frame *k*
*η*	The ratio between the weight/height of the object at the first frame
*ε*	Epsilon
*Δx*	Dirac delta function at *x*
Δ	Kronecker function
*N*(0, *σ*_*x*_^2^)	Gaussian transition function
HOG	Histogram of oriented gradients
*N*_eff_	Effective sample size

## Data Availability

Public data were used to research. The [TB-100] data used to support the findings of this study are included within the article.

## Appendix

Table 2 describes the notations.

### A. Sample to Train Gentle Adaboost

Figure 8 describes the random true-positive sampling with the bordering error at ±5 pixels and false-positive sampling which does not include any particular object.
